# The Effect of Formulation of Curcuminoids on Their Metabolism by Human Colonic Microbiota

**DOI:** 10.3390/molecules25040940

**Published:** 2020-02-19

**Authors:** Letizia Bresciani, Claudia Favari, Luca Calani, Veronica Francinelli, Antonella Riva, Giovanna Petrangolini, Pietro Allegrini, Pedro Mena, Daniele Del Rio

**Affiliations:** 1 Department of Veterinary Science, University of Parma, 43126 Parma, Italy; letizia.bresciani@unipr.it; 2 Department of Food and Drugs, University of Parma, 43124 Parma, Italy; claudia.favari@studenti.unipr.it (C.F.); luca.calani@unipr.it (L.C.); veronica.francinelli@studenti.unipr.it (V.F.); pedromiguel.menaparreno@unipr.it (P.M.); 3 Research and Development Department, Indena S.p.A., 20139 Milano, Italy; antonella.riva@indena.com (A.R.); giovanna.petrangolini@indena.com (G.P.); pietro.allegrini@indena.com (P.A.)

**Keywords:** curcuminoids, turmeric, curcumin, phytosome, colonic metabolism

## Abstract

Turmeric (*Curcuma longa* L.) is the only edible plant recognized as a dietary source of curcuminoids, among which curcumin, demethoxycurcumin (DMC) and bis-demethoxycurcumin (Bis-DMC) are the most representative ones. Curcumin shows a very low systemic bioavailability and for this reason, several technologies have been adopted to improve it. These technologies generally improve curcuminoid absorption in the small intestine, however, no data are available about the effect of curcuminoid formulation on colonic biotransformation. The present study aims at investigating the human colonic metabolism of curcuminoids, prepared with two different technologies, using an in vitro model. Unformulated curcuminoid and lecithin-curcuminoid botanical extracts were fermented using an in vitro fecal model and colonic catabolites were identified and quantified by uHPLC-MS^n^. Native compounds, mainly curcumin, DMC and bis-DMC, were metabolized by colonic microbiota within the 24-h incubation. The degradation of curcuminoids led to the formation of specific curcuminoid metabolites, among which higher concentrations of bis(demethyl)-tetrahydrocurcumin and bis(demethyl)-hexahydrocurcumin were found after lecithin-extract fermentation compared to the concentration detected after unformulated extract. In conclusion, both curcumin-based botanical extracts can be considered important sources of curcuminoids, although the lecithin-formulated extract led to a higher production of curcuminoid catabolites. Moreover, a new curcuminoid catabolite, namely bis(demethyl)-hexahydrocurcumin, has been putatively identified, opening new perspectives in the investigation of curcuminoid bioavailability and their potential metabolite bioactivity.

## 1. Introduction

Turmeric (*Curcuma longa* L.) is the only edible plant recognized as a dietary source of curcuminoids, among which curcumin is the most abundant compound, followed by demethoxycurcumin (DMC) and bis-demethoxycurcumin (Bis-DMC). These compounds are yellow-orange non-polar polyphenols, made of two aromatic rings joined by a seven-carbon alkyl chain that contains two ketone groups. The interest of the scientific community on curcumin increased in the last decade [1] because of its putative bioactivity in humans [2,3,4,5]. However, curcumin shows low oral absorption, little biodistribution, and a very low systemic bioavailability. For these reasons, several technologies and different formulations have been investigated to improve the bioavailability of this diarylheptanoid compound. The nano-particulate forms of curcumin increase its oral bioavailability through different formulations, such as poly(2-hydroxyethyl methacrylate), cyclodextrin, silica, casein, chitosan, and glyceryl monoleate nanoparticles [6]. Other technologies, including phytosomes, liposomes, and micelles, besides the curcumin co-formulation with adjuvants, like piperine, have also been adopted to enhance the bioavailability of curcumin [6]. Although these technologies and formulations improve the curcuminoid absorption in the small intestine, a large fraction of curcuminoids generally reaches the colon and is excreted via feces, as a favorite elimination route [7]. Several studies have reported an important role of curcumin on gut microbiota modulation [1,8,9,10] and, as a consequence, the investigation of the interaction between curcuminoids and gut microbiota has gained more relevance.

To date, no information is available on the effect of curcuminoid formulation and delivery system on the catabolic biotransformation carried out by the human colonic microbiota. This step represents a crucial point since the human microbiota has been reported to perform several metabolic/catabolic reactions towards curcuminoids, which should be taken into account in the overall investigation of curcumin bioactivity and bioavailability [11,12,13]. More in-depth studies on the colonic bioconversion of curcuminoids are needed to unravel the possible pool of catabolites derived from curcuminoids colonic metabolism [14,15,16].

Thus, the aim of the current study was to describe the interaction and the human colonic metabolism of curcuminoids, prepared with two different technologies (unformulated and lecithin-formulated botanical extracts), using an in vitro fecal fermentation model.

## 2. Results

### 2.1. Curcuminoid Characterization in Botanical Extracts

Both unformulated and lecithin-formulated botanical extracts shared the same curcuminoid profile, with curcumin, demethoxycurcumin (DMC), and Bis-DMC being the main compounds, while dihydrocurcumin and hydroxycurcumin were minor diarylheptanoids. The curcuminoid concentrations in both curcumin-derived botanical extracts are detailed in Table 1. Curcumin, DMC and Bis-DMC showed a double chromatographic peak because of the keto-enol tautomerism, as previously reported for curcuminoids [11,17,18,19]. Dihydrocurcumin was identified based on the mass spectral characteristics previously reported [20,21]. Finally, hydroxycurcumin was identified comparing its chromatographic behavior with that of curcumin and through MS^n^ ion spectra comparison. In particular, hydroxycurcumin identification was based on MS^3^ experiments, obtained by fragmentation of MS^2^ ion peak at *m*/*z* 217, which resulted in the same MS^3^ fragmentation pattern of curcumin, having a product ion at *m*/*z* 173 (Table 2). The curcumin concentration, obtained by the sum of both chromatographic peaks, was about 94.3% in unformulated botanical extract and 32.4% for lecithin-curcuminoid botanical extract (Table 1). Curcumin was the only curcuminoid quantified with a reference standard. Consequently, this approach may lead to misleading results related to an inaccurate semi-quantification, which may occur when quantifying with their structurally-related phenolic compounds for which reference standards are not commercially available [22]. Nevertheless, this common limitation does not condition the comparison between the two curcumin-derived botanical extracts.

### 2.2. Colonic In Vitro Biotransformation of Curcuminoids

**Figure 1:**
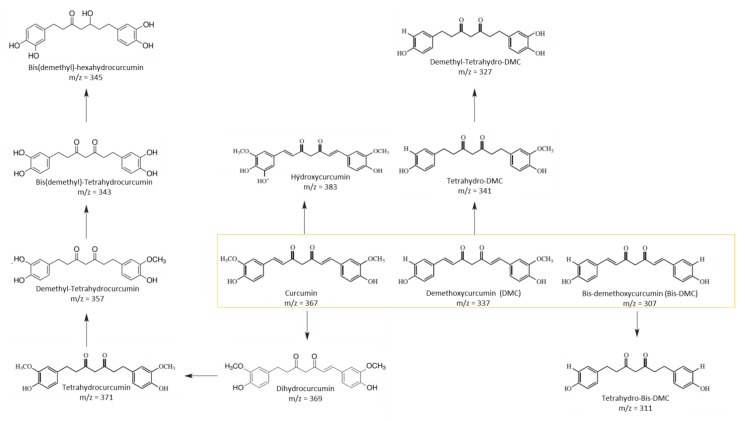
Proposed microbial conversion of native curcuminoids in their fecal metabolites.

Seven curcuminoid metabolites were tentatively identified through their chromatographic behavior and based on their mass spectral characteristics (Table 2). No microbial-derived compounds were recovered at baseline, as well as in the control fecal slurries without curcuminoid incubation, at 5 h and 24 h. Moreover, no microbial metabolites were detected after 5 h and 24 h of curcuminoid incubation with culture medium deprived of fecal slurry, confirming that the appearance of microbial-derived curcuminoid metabolites is strictly related to the interaction of parent compounds with fecal microbiota. Despite the higher number of identified curcuminoid metabolites in the present work with respect to what we previously identified [11], dihydroferulic acid and 1-(4-hydroxy-3-methoxyphenyl)-2-propanol were not detected. Most of the detected fecal metabolites were principally derived from the reduction of the double bonds of curcuminoids, as well as from the demethylation or (bis)demethylation of curcuminoids, as previously observed after incubation with human colonic microbiota (Figure 1) [11,13,23]. The most polar metabolite, with a retention time of around 4.0 min, showed a deprotonated molecular ion at *m*/*z* 345, yielding fragment ions at *m/z* 165 and 179, revealing a structure close to that of hexahydrocurcumin [20,24], which might correspond to bis(demethyl)hexahydrocurcumin. In general, most of the colonic metabolites recovered after fecal fermentation of the unformulated botanical extract and lecithin-formulated botanical extract showed double chromatographic peaks because of the rapid transition between the keto-enol/β-diketone structures related to keto-enol tautomerism, as it occurs for plant-derived curcuminoids [11,17,18,19].

Table 3 shows the concentration of native curcuminoids and their microbial metabolites in fecal slurries at different incubation periods. Curcumin quantified at 0 h was found to be the same both in unformulated- and formulated fermented batches, confirming that the same amount of curcumin was added. On the contrary, since the extract amount was standardized for curcumin content, DMC, bis-DMC, dihydrocurcumin and hydroxycurcumin were found to be significantly higher in those batches containing lecithin-curcuminoid botanical extract (Table 3).

All the curcuminoid metabolites in fecal batches were semi-quantified as curcumin equivalents, due to the unavailability of their reference standard compounds and to compare the concentrations of both parent curcuminoids and their microbial metabolites in unformulated and formulated botanical extracts. Although all metabolites appeared after 5 h of fermentation, most of them reached their maximum concentration after 24 h incubation. The highest concentrations were recorded for demethylated metabolites, which could explain the drop observed for parent curcuminoids. The decrease of curcumin after 24 h of fecal fermentation corresponded to 22.7% and 63.5% of curcumin quantified at the beginning of the fermentation (0 h) for unformulated- and formulated botanical extract, respectively. These percentages increased when the total native curcuminoids were considered, with a decrease of 24.6% and 72.0% for unformulated-curcuminoid botanical extract and lecithin-curcuminoid botanical extract, respectively. Taking into account all the microbial-derived metabolites detected at 24 h, the production of colonic metabolites was 2.7-fold higher in the lecithin-formulated extract (413.5 and 153.6 μmol/L, respectively) with respect to the unformulated curcuminoid extract. Nevertheless, it should be noted again that these data are semi-quantitative. Consequently, differences between products are relative and a mass balance between parent and colonic compounds cannot be performed.

The robustness of this in vitro fermentation model was assessed through uHPLC-MS^n^ analysis of parent curcuminoids incubated at different time points with bacterial culture medium without feces. All parent curcuminoids were chemically stable in these conditions, especially for the lecithin formulation. Curcumin in the unformulated botanical extract was recovered at micromolar concentrations equal to 173.2, 129.4, and 149.4 at baseline, 5 h, and 24 h, respectively. Curcumin in the lecithin-formulated botanical extract was recovered at micromolar concentrations equal to 214.9, 212.9, and 202.0 at baseline, 5 h, and 24 h, respectively.

## 3. Discussion

The present study provides new information on metabolism by human fecal bacteria towards turmeric curcuminoids depending on their formulation. Although no difference in the type of metabolites emerged between the lecithin-curcuminoid botanical extract and the unformulated botanical extract from a qualitative point of view, the phospholipid formulation led to a higher microbial degradation of parent compounds than the unformulated curcumin extract, with a prominent drop of native turmeric curcuminoids after 24 h-incubation. Thus, the curcuminoids in the phospholipid delivery system, which was developed as a solid-state dispersion of curcumin, underwent a more efficient microbial biotransformation than in the simple curcuminoid extract. These data show an opposite trend with respect to a previous work, which stated that lecithin-derived curcuminoids were more resistant to the catabolic action of colonic microbiota [25]. Previously, Papillo and colleagues (2019) investigated curcuminoid degradation up to 5 h. The present study by comparing the lecithin and simple botanical extract formulation showed the differences in metabolite formation mainly occurred after 24 h of fecal incubation. Considering only the first incubation period (5 h), curcumin, DMC and bis-DMC were generally poorly metabolized by colonic microbiota when incubated as an unformulated botanical extract, whereas lecithin-curcuminoids DMC and bis-DMC slightly decreased already within 5 h, but curcumin remained stable during the first incubation period. Among the other minor parent curcuminoids, dihydrocurcumin increased after 5 h of incubation, both in formulated and unformulated curcuminoids, probably due to the transient intermediate metabolite of curcumin involved in the formation of tetrahydrocurcumin [26]. Dihydrocurcumin decreased till the end of the incubation, while hydroxycurcumin increased after 5 h fermentation, both in formulated and unformulated curcuminoid extract, suggesting a putative hydroxylation reaction towards curcumin, a common microbial metabolic step [13]. The microbial catabolic pathway of curcuminoids is proposed in Figure 1. After the first fecal incubation period (5 h), curcuminoid catabolites were mainly recovered as tetrahydro-forms, because of the action of microbial reductases. In contrast, parent curcuminoids were largely metabolized by colonic microbiota within 24 h, resulting in the demethylation and/or bis(demethylation) of the tetrahydro-forms of parent curcuminoids. Bis(demethyl)-tetrahydrocurcumin, bis(demethyl)-hexahydrocurcumin and demethyl-tetrahydro-DMC were the most abundant curcuminoid catabolites produced after 24 h of fecal fermentation, and these compounds were significantly higher in the phospholipid formulation.

To the best of our knowledge, the present work reports, for the first time, bis(demethyl)-hexahydrocurcumin (*m*/*z* 345) among the main microbial-derived curcuminoid catabolites. Its identification has been performed based on its mass spectral characteristics and a mass fragmentation behavior close to that of hexahydrocurcumin [20,24]. These findings are in agreement with previous works that unraveled the human colonic microbiota as able to produce demethylated-curcuminoids [12,13].

Previous studies have shown that phospholipid delivered curcuminoids are better absorbed in the upper intestinal tract than curcuminoids in their native unformulated form [27,28]. However, no data were available about the effect of formulation on curcuminoid microbial catabolism. The new information showed in the present study reinforces the crucial role of colonic microbiota towards curcuminoid catabolism, as it occurs for other polyphenols [14]. Furthermore, the phospholipid formulation does not give rise to new metabolites by the fermentation assay but resulted in a more efficient biotransformation of curcumin in respect to the unformulated extract. Lastly, these findings could be useful to reconsider the overall bioavailability of turmeric-derived curcuminoids and to better understand the putative bioactivity ascribed to curcuminoids, mainly curcumin, through in vivo studies.

## 4. Materials and Methods

### 4.1. Chemicals

Curcumin powder, with the purity specification equal to 80.1%, as previously reported [11], acetone, isopropanol, methanol, formic acid, bile salts, calcium chloride (CaCl_2_), (+)-arabinogalactan, tryptone, yeast extract, buffered peptone water, Dulbecco’s phosphate buffer saline, casein sodium salt from bovine milk, pectin from citrus fruits, mucin from porcine stomach-type III, NaHCO_3_, KH_2_PO_4_, MgSO_4_ monohydrate, guar gum, inulin, Tween 80, xylan from birchwood, L-cysteine hydrochloride monohydrate, FeSO_4_ heptahydrate, and resazurin redox indicator were obtained from Sigma-Aldrich (St. Louis, MO, USA). Anhydrous K_2_HPO_4_ and soluble starch were purchased from Carlo Erba Reagents (Milan, Italy). KCl and NaCl were obtained from Merck (Darmstadt, Germany). Both HPLC-grade water and HPLC-grade acetonitrile were purchased from VWR International (Milan, Italy).

### 4.2. Curcumin-Based Products

Both Curcumin 95% (unformulated botanical extract) and Curcumin Phytosome (lecithin-formulated botanical extract, MERIVA^®^) were provided by Indena S.p.A. (Milan, Italy). Prior uHPLC-MS^n^ analysis, the unformulated extract was dissolved in pure methanol (4 mL), while lecithin-formulated extract required a mixture of acetone/isopropanol/methanol 50:33.33:16.66 (*v*/*v*/*v*) for a better extract dissolution. Both solutions were then adequately diluted with aqueous acetonitrile, acidified with formic acid (60:39.8:0.2, *v*/*v*) before uHPLC-MS^n^ analyses.

### 4.3. In Vitro Fecal Fermentation

The culture medium, as well as the human fecal slurry, were prepared as previously reported in in vitro fecal experiments of polyphenol-rich products and curcuminoids [11,29]. The extracts used for the in vitro fermentation have been suspended in water, vortexed 5 min, and sonicated 10 min. The sonication (5 min) and the vortex steps (5 min) were repeated on the experiment day, prior to adding the sample suspension to the fermentation batch. The composition of 1 L of growth medium was 2.5 g of soluble starch, 2.5 g of peptone, 2.5 g of tryptone, 2.25 g of yeast extract, 2.25 g of NaCl, 2.25 g of KCl, 1 g of pectin, 2 g of mucin, 1.5 g of casein, 1 g of arabinogalactan, 0.75 g of NaHCO_3_, 0.35 g of MgSO_4_·H_2_O, 0.5 g of guar gum, 0.5 g inulin, 1 g of xylan, 0.4 g of l-cysteine HCl·H_2_O, 0.25 g of KH_2_PO_4_, 0.25 g of K_2_HPO_4_, 0.2 g of bile salt, 0.04 g of CaCl_2_, 0.0025 g of FeSO_4_·7H_2_O, 0.5 mL of Tween 80, and 2 mL of resazurin solution (0.025%, *w*/*v*) as an anaerobic indicator. The growth medium was sterilized at 121 °C for 15 min in glass vessels (12 mL) before sample preparation. Fresh fecal slurries were collected from three healthy volunteers who did not have previously any intestinal disease and were not treated with antibiotics for the previous 3 months [30]. After collection, fecal slurries were immediately stored in an anaerobic jar and processed within 2 h. Feces from donors were pooled and then diluted with sterilized Dulbecco’s phosphate buffer saline at 1% (*w*/*v*) and homogenized to obtain a 10% (*w*/*w*) slurry to be used as the fermentation starter [30]. In each fermentation batch, 45% of the growth medium, 45% of fecal slurry, and 10% of extract suspension (final concentration of curcumin 300 μmol/L) were added to reach a total fermentation volume of 4 mL. The fermentation starter and the samples were introduced in the vessel containing sterilized growth medium, sealed with a rubber seal, and flushed through a double-needle with nitrogen to create anaerobic conditions. Vessels were then incubated for 24 h at 37 °C at 200 strokes per min in a Dubnoff bath (ISCO, Milan, Italy) and collected after 0, 5 and 24 h. Following incubation, microbial metabolism was stopped by adding 10% of acetonitrile to 4 mL of fermented sample and samples were frozen (−18 °C) until extraction and analysis. All experiments were carried out in triplicate.

### 4.4. Fecal Metabolite Extraction

Curcuminoids and their metabolites from fermented fecal slurries were extracted following the method reported by Tan et al. [11], with slight modifications. Briefly, these compounds were extracted by adding 1 mL of ethyl acetate, vortexed for 1 min, and sonicated for 10 min in an ultrasonic bath. The upper organic layer (900 µL) was transferred to a clean microfuge tube. The above extraction procedure was repeated once using just the lower layer and transferring 1000 µL in a clean microfuge tube. Both upper layers were dried for about 2 h at room temperature through a centrifugal vacuum concentrator (SpeedVac Savant SPD121P, Thermo Fisher Scientific Inc., San Jose, CA, USA). The residue was then reconstituted in 200 µL of aqueous acetonitrile, acidified with formic acid (60:39.8:0.2, *v*/*v*), vortexed, sonicated for 5 min and centrifuged at 14,462× *g* for 5 min before uHPLC-MSn analyses.

### 4.5. uHPLC/MS^n^ Analysis

Ultra-high performance liquid chromatography coupled to a linear ion trap mass spectrometer (uHPLC-LIT-MS) was used both for curcuminoid quantification in the formulated and unformulated botanical extracts and for the analysis of curcuminoid degradation and metabolite formation in the human fecal fermentations. An Accela uHPLC 1250 equipped with a LIT-MS (LTQ XL, Thermo Fisher Scientific Inc., San Jose, CA) fitted with a heated ESI probe (H-ESI-II, Thermo Fisher Scientific Inc.) was used for all the analysis. Curcumin extracts were characterized using full scan, data-dependent, MS^3^ experiments, monitoring from *m*/*z* 100 to 1000, while the analysis of the microbial degradation of curcuminoids was carried out in full scan, data-dependent, MS^2^ experiments, monitoring *m*/*z* from 100 to 500. All curcuminoids and their fecal metabolites were semi-quantified as curcumin (purity 80%) through external calibration curves, by extracting the corresponding *m*/*z* from the full scan chromatograms. The MS response of curcumin was linear in the range of 1–200 µM, while both intra-day precision and inter-day precision were below 10% (measured as the relative standard deviation of the slopes obtained by different calibration curves). The limit of detection was equal to 0.5 µM (signal to noise S/N ≥ 3) while the limit of quantification was 1 µM (S/N ≥ 10). Further targeted MS^n^ analyses were carried out to pinpoint the appearance time of all metabolites, as well as to get more information about curcuminoid metabolites. The MS worked in negative ionization mode, with a capillary temperature of 275 °C, while the source was maintained at 250 °C. The sheath gas (N_2_) flow was 40 units, while auxiliary and sweep gases (N_2_) were set to 5 units. The source voltage was 3 kV. The capillary voltage and tube lens were at −5 and −68 V, respectively. Ultra-pure helium gas (99.9999%) was used in MS^n^ analysis with a collision-induced dissociation (CID) equal to 35. For uHPLC, mobile phase A was 0.1% (*v/v*) formic acid in water and mobile phase B was 0.1% (*v*/*v*) formic acid in acetonitrile. Separations were carried out by means of a Kinetex Evo C18 column (100 × 2.1 mm; 2.6 µm particle size; Pheneomenex, CA, USA) installed with a precolumn cartridge (Phenomenex). The mobile phases, pumped at a flow rate of 0.4 mL/min, were kept at 10% B for 1 min, and then B turned up to 80% in 10 min keeping this value for 3 min, followed by a 4 min re-equilibration time at the start conditions. Chromatograms and mass spectral data were acquired using Xcalibur software 2.1 (Thermo Fisher Scientific Inc., San Jose, CA, USA).

### 4.6. Statistical Analysis

Experiments were carried out in triplicate. Results are shown as the mean ± standard deviation (SD). T-test was applied to detect significant mean differences between products for each time point. ANOVA (Bonferroni post hoc test for independent samples) was used to verify differences in compound production when different incubation periods (0–5–24) were compared using the same botanical extract (*p* < 0.05). All statistical analyses were performed using the SPSS statistical package (version 25, SPSS, Inc., Chicago, IL, USA).

## 5. Conclusions

Both curcumin-based botanical extracts (unformulated and lecithin-formulated) can be considered important sources of curcuminoids. The three major curcuminoids quantified in the formulated and unformulated extracts were catabolized in vitro by fecal microbiota leading to the formation of several microbial-derived catabolites. The formulation notably influenced the biotransformation of curcuminoids, since the fermentation of lecithin-formulated curcuminoids led to more efficient production of curcuminoid catabolites. Finally, a new curcuminoid catabolite, namely bis(demethyl)-hexahydrocurcumin, has been tentatively identified and semi-quantified after curcuminoid in vitro biotransformation, opening new perspectives in the investigation of turmeric-derived curcuminoid bioavailability and their potential metabolite bioactivity.

## Figures and Tables

**Table 1:** Quantification of curcuminoids in curcumin-derived botanical extracts. Data are expressed as mg/g (mean values ± SD, *n* = 3).

Compound	Unformulated Botanical Extract	Lecithin-Formulated Botanical Extract
Curcumin	943.2 ± 4.4	324.4 ± 5.3
DMC	332.1 ± 5.3	148.8 ± 3.3
Bis-DMC	37.6 ± 0.6	22.0 ± 0.7
Dihydrocurcumin	12.7 ± 0.6	4.6 ± 0.9
Hydroxycurcumin	4.4 ± 0.1	4.6 ± 0.2

**Table 2:** Mass spectrometric and chromatographic characteristics of parent curcuminoids and their fecal metabolites identified in curcumin botanical extracts and in fecal fermented samples.

Compound	RT	[M − H]^−^ (*m*/*z*)	MS^2^	MS^3^
Curcumin	5.9–7.6	367	**217**, 173, 149, 175	173, 202
DMC	5.8–7.4	337	**217**, 187, 173, 143	173, 202
Bis-DMC	5.7–7.3	307	**187**, 143, 119	143
Dihydrocurcumin	7.3	369	219, 149, 233, 175, 149, 134	
Hydroxycurcumin	6.7	383	**217**, 173, 165, 233, 150	173, 202
Tetrahydrocurcumin	5.9–7.1	371	235, 249, 247, 207, 193, 121	
Demethyl-Tetrahydrocurcumin	5.2–6.4	357	235, 247, 193, 121, 109	
Bis(demethyl)-Tetrahydrocurcumin	4.5–5.6	343	221, 233, 203, 121, 163	
Tetrahydro-DMC	5.8–7.0	341	219, 235, 205, 247, 121, 177	
Tetrahydro-Bis-DMC	5.7–6.9	311	205, 217, 269	
Demethyl-Tetrahydro-DMC	5.1–6.3	327	205, 217, 221, 109, 99, 121	
Bis(demethyl)hexahydrocurcumin	4.0	345	**165**, **179**, 121, 223, 109, 235, 205	165: 121, 109, 123; 179: 121, 109, 57

DMC: demethoxycurcumin; Bis-DMC: bis-demethoxycurcumin. MS^2^ ions in bold were those subjected to MS^3^ fragmentation. The two retention times (RT) account for the presence of keto-enol tautomers.

**Table 3:** Concentration (μmol/L) of native curcuminoids and their faecal metabolites at different time points.

Compound	Unformulated Botanical Extract			Lecithin-Formulated Botanical Extract		
	0 h	5 h	24 h	0 h	5 h	24 h
**Curcumin**	158.3 ± 25.3 ^a^	166.1 ± 15.9 ^a^	122.3 ± 8.5 ^a^	207.9 ± 30.4 ^a^	211.3 ± 8.9 ^a,^*	75.9 ± 6.3 ^b,^**
**DMC**	45.7 ± 10.8 ^a^	48.4 ± 6.9 ^a^	32.4 ± 3.5 ^a^	85.4 ± 12.7 ^a,^*	54.1 ± 2.8 ^b^	9.7 ± 1.3 ^c,^***
**Bis-DMC**	5.0 ± 1.2 ^a^	4.9 ± 0.7 ^a^	2.8 ± 0.3 ^a^	15.1 ± 2.4 ^a,^**	10.4 ± 0.4 ^b,^***	1.7 ± 0.2 ^c,^**
**Dihydrocurcumin**	2.5 ± 0.5 ^ab^	3.3 ± 0.5 ^a^	1.6 ± 0.1 ^b^	6.4 ± 0.8 ^a,^**	8.8 ± 0.5 ^b,^***	0.6 ± 0.1 ^c,^***
**Hydroxycurcumin**	0.4 ± 0.1 ^a^	0.9 ± 0.2 ^b^	0.6 ± 0.1 ^a^	4.2 ± 0.8 ^a,^*	5.8 ± 0.5 ^b,^***	1.3 ± 0.2 ^c,^**
**Tetrahydrocurcumin**	n.d.	4.4 ± 0.1	n.d.	n.d.	12.1 ± 0.5 **	n.d.
**Demethyl-Tetrahydrocurcumin**	n.d.	14.2 ± 0.9	n.d.	n.d.	15.9 ± 3.1 ^a^	0.6 ± 0.1 ^b,^***
**Bis(demethyl)-Tetrahydrocurcumin**	n.d.	3.9 ± 0.9 ^a^	74.6 ± 5.4 ^b^	n.d.	0.9 ± 0.3 ^a,^**	235.9 ± 5.6 ^b,^***
**Tetrahydro-DMC**	n.d.	3.4 ± 0.1 ^a^	0.4 ± 0.0 ^b^	n.d.	19.6 ± 1.2 ^a,^**	2.0 ± 0.3 ^b,^**
**Tetrahydro-Bis-DMC**	n.d.	1.4 ± 0.2 ^a^	1.9 ± 0.1 ^b^	n.d.	3.3 ± 0.3 ^a,^**	7.7 ± 0.0 ^b,^***
**Demethyl-Tetrahydro-DMC**	n.d.	1.7 ± 0.3 ^a^	12.9 ± 1.0 ^b^	n.d.	5.4 ± 1.0 ^a,^**	52.9 ± 1.3 ^b,^***
**Bis(demethyl)-hexahydrocurcumin**	n.d.	1.3 ± 0.2 ^a^	63.8 ± 4.5 ^b^	n.d.	0.8 ± 0.2 ^a^	114.4 ± 0.9 ^b,^***

Data are expressed as mean ± SD (*n* = 3). Different letters indicate significant differences considering the same botanical extract, the same compound, but different incubation periods (*p* < 0.05). * *p* < 0.05; ** *p* < 0.01; *** *p* < 0.001, significant differences between products comparing the same incubation period (0 h, 5 h and 24 h). n.d.: not detected.
